# From Straw to Device Interface: Carboxymethyl‐Cellulose‐Based Modified Interlayer for Enhanced Power Conversion Efficiency of Organic Solar Cells

**DOI:** 10.1002/advs.201902269

**Published:** 2019-11-27

**Authors:** Junying Wu, Yanjun Liu, Amjad Islam, Qinghong Zheng, Jianguo Li, Wei Ji, Lihui Chen, Xinhua Ouyang

**Affiliations:** ^1^ College of Materials Engineering Fujian Agriculture and Forestry University Fuzhou 350000 P. R. China; ^2^ Department of Physics National University of Singapore 2 Science Drive 3 Singapore 117542 Republic of Singapore

**Keywords:** carboxymethyl cellulose, interface layers, organic solar cells, straw

## Abstract

Advanced interface materials made from petrochemical resources have been extensively investigated for organic solar cells (OSCs) over the past decades. These interface materials have demonstrated excellent performances in OSC devices. However, the limited resources, high‐cost, and non‐ecofriendly nature of petrochemical‐based interface materials restrict their commercial applications. Here, a facile and effective approach to prepare cellulose and its derivatives as a cathode interface layer for OSCs with enhanced performance from rice straw of agroforestry residues is demonstrated. By employing this carboxymethyl cellulose sodium (CMC) into OSCs, a highly efficient inverted OSC is constructed, and a power conversion efficiency (PCE) of 12.01% is realized using poly[(2,6‐(4,8‐bis(5‐(2‐ethyl‐hexyl)‐thiophen‐2‐yl)‐benzo[1,2‐b:4,5‐b′] dithiophene))‐*alt*‐(5,5‐(1′,3′‐di‐2‐thienyl‐5′,7‐bis(2‐ethylhexyl)benzo[1′,2′‐c: 4′,5′‐c′]dithiophene‐4,8‐dione): 3,9‐bis(2‐methylene‐((3‐(1, 1‐dicyanomethylene)‐6/7‐methyl)‐indanone))‐5,5,11,11‐tetrakis(4‐hexylphenyl)‐dithieno[2,3‐d: 2′,3′‐d′]‐s‐indaceno[1,2‐b: 5, 6‐b′]dithiophene as the active layer, which shows over 9.4% improvement in PCE compared to that of a device without the CMC layer (PCE = 10.98%), especially the enhancement in short‐circuit current. The improved current densities and PCEs are attributed to the reduced work function, enhanced absorption, and improved interfacial contact by using CMC and ZnO as co‐interface. This approach of fabricating interface materials from biorenewable sources for OSCs is simple, scalable, and cost‐effective, representing a promising direction for the development of smart interface and green electronics.

## Graphical abstract

An effective approach to prepare cellulose as interface of organic solar cells (OSCs) with enhanced performance from rice straw of agroforestry residues is demonstrated. A highly efficient inverted OSC is constructed and a power conversion efficiency (PCE) of 12.01% is realized using PBDB‐T:IT‐M as the active layer, which shows over 9.4% improvement in the PCE compared to that of a counterpart device (PCE = 10.98%).

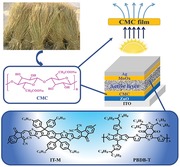

The research on organic solar cells (OSCs) has made an immense progress over the last one decade because OSCs possess multiple advantages, such as light‐weight, efficient, economical and large‐area fabrication.^1^, ^2^, ^3^ To date, the power conversion efficiency (PCE) at the laboratory scale has reached up to ≈16% by employing novel interfacial and photoactive materials.^4^ Prior to the importance of photoactive layer, interfacial layer also plays a vital role to fabricate high‐performance OSC by reducing the energy barrier at the interface and improving charge transfer.^5^, ^6^ However, it is usually impossible for the conventional single interlayer materials to satisfy all the requirements. In this regard, it is highly desirable to develop novel interfacial materials with suitable modification for highly efficient OSCs.

To develop effective cathode interfacial materials, interface barrier should be minimized with improved electron mobility. Generally, interface barrier originates directly from the mismatch of the energy levels between the active layer and electrodes. Therefore, the key is to design suitable interfacial materials which can significantly reduce the interface barrier between the electrodes and active layer. In the past decades, numerous attempts have been made and significant progresses have been achieved for the cathode interface modification of OSCs. For example, few metallic salts, including LiF, CsF, and Cs_2_CO_3_,^7^, ^8^, ^9^ have been integrated into OSCs to modify the interface and the devices exhibited enhanced efficiencies. However, the electron transfer ability of these metallic salts is low and these layers are thickness sensitive (<1 nm), which restrict their application on large scale. In this regard, some organic materials are also introduced for the interface modification of OSCs. Generally, organic materials are synthesized easily with high electron mobility and tunable bandgap energy. Some of them (poly[(9,9‐bis(3′‐(*N*,*N*‐dimethylamino)propyl)‐2,7‐fluorene)‐*alt*‐2,7‐(9,9‐dioctyfluorene)], polyethylenimine, and polyethylenimine ethoxylated (PEIE)) were found to be effective interface modifiers with matched performance of these devices, compared to the devices using conventional interlayer materials (Ca/Mg).^10^, ^11^ However, these organic electrolytes have complicated structures with difficult synthetic procedure. Moreover, some of them show poor electron transporting ability. Therefore, the design and synthesis of novel interface materials with simple structures and excellent charge transporting abilities are still considered to be a challenging task. A couple of years ago, our group reported couple of nonconjugated small‐molecule electrolyte cathode interlayers such as (4,4′‐(((methyl(4‐sulfonatobutyl)ammonio)bis(propane‐3,1‐diyl)) bis(dimethyl‐ammoniumdiyl)) bis‐(butane‐1‐sulfonate) (MSAPBS) and 4,4′‐((oxybis(ethane‐ 2, 1‐diyl)) bis(dimethyl‐ammoniumdiyl)) bis(butane1‐sulfonate) for OSCs which showed significant improvement in PCE of the devices.^12^, ^13^ Although these nonconjugated small‐molecule electrolytes are synthesized with simple procedure and improved electron mobility, but they are prepared from petroleum‐based materials, which present serious environmental concerns. In this regard, it is highly desirable to develop novel, cost‐effective, and ecofriendly biomass‐based efficient interfacial materials for OSCs.

Cellulose and its derivatives are one of the promising natural materials due to their abundant, biorenewable, biodegradable and environmentally friendly characteristics.^14^, ^15^, ^16^ Importantly, these cellulose and derivatives have some similar moieties with PEIE and MSAPBS, which can emerge as potential materials for the interface layer of OSC devices. Very recently, cellulose has been used as counter electrode for dye‐sensitized solar cells^17^ and flexible substrates for perovskite solar cells^18^ for green electronics by our group. Carboxymethyl cellulose sodium (CMC), one of the typical cellulose derivatives, can be prepared from rice straw and reed of agroforestry residues,^19^ and has been applied for different functions such as adhesive, stabilizer, and film former.^20^ However, to the best of our knowledge, the use of CMC for highly value‐added applications is still very limited, especially for the interface material of OSCs.

Herein, we report the first time application of CMC prepared from rice straws to be used as cathode interlayer for OSCs in inverted structure, thus realizing the real meaning of “turning straw into gold.” In these devices, CMC is used as a comodifying layer with zinc oxide (ZnO) for the transfer and collection of electrons. A blend of poly[(2,6‐(4,8‐bis(5‐(2‐ethyl‐hexyl)‐thiophen‐2‐yl)‐benzo[1,2‐b:4,5‐b′] dithiophene))‐*alt*‐(5,5‐(1′,3′‐di‐2‐thienyl‐5′,7‐bis(2‐ethylhexyl)benzo[1′,2′‐c: 4′,5′‐c′]dithiophene‐4,8‐dione)] (PBDB‐T) and 3,9‐bis(2‐methylene‐((3‐(1, 1‐dicyanomethylene)‐6/7‐methyl)‐indanone))‐5,5,11,11‐tetrakis(4‐hexylphenyl)‐dithieno[2,3‐d: 2′,3′‐d′]‐s‐indaceno[1,2‐b: 5, 6‐b′]dithiophene (IT‐M) is employed as the photoactive layer (the device and chemical structures are shown in **Scheme** 1). By spin‐coating a layer of CMC onto ZnO, the PCE of OSCs can be enhanced to 12.01%, which is ≈9.4% higher than that of pristine ZnO‐based OSCs with the efficiency of 10.98% (inverted devices) by using the same active layer. The improved current densities and PCEs are attributed to the reduction in work function (WF), increased absorption, and better interfacial contact by using CMC and ZnO as co‐interface. The obtained results create a new pathway for achieving highly efficient OSCs using environment friendly biomass materials. More importantly, these findings also provide a strategy to efficiently utilize agricultural and forestry residues.

**Scheme 1:**
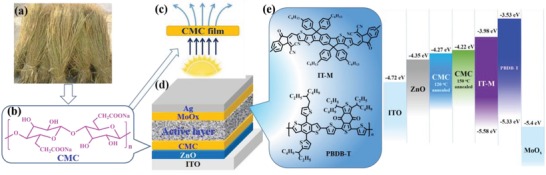
a) Schematic of carboxymethyl cellulose sodium (CMC) from straw. b) The molecular structure of CMC. c) Schematic diagram of CMC layer and its light scattering behavior. d) The device construction of organic solar cells. e) The molecular structures of active layer materials using for the cells. f) The diagram of energy levels of device materials.

In order to tune the energy barrier between indium‐tin oxide (ITO) and active layer materials, double layer structure of ZnO and CMC was introduced. The experimental measurement of WF was carried out in an electron spectroscopy for chemical analysis (ESCA) system of an X‐ray photoelectron spectrometer with the vacuum value of 5 × 10^−8^ Pa. Ultraviolet photoelectron spectroscopy (UPS) was employed with He I (*h*_v_ = 21. 22 eV), sample was added with −5 V bias. Before the measurement, the sample was cleaned by Ar ion sputter. As shown in **Figure** 1a, it can be seen that the WFs of ITO and ITO/ZnO are ≈4.72 eV and ≈4.35 eV, respectively, which is in good agreement with the reported results.^21^ For the ITO with ZnO and CMC layer (120 °C annealed), reduced WF was observed with a value of 4.27 eV. The lower WF of ZnO/CMC will be beneficial to achieve ohmic contact with the IT‐M acceptor and ITO electrode. Moreover, this will improve the electron extraction efficiency and decrease recombination losses. Likewise, the film ZnO/CMC annealed at 150 °C exhibited similar WF with that of ZnO/CMC (120 °C annealed), the value is ≈4.22 eV. The results from UPS showed that the deposition of CMC onto ZnO will effectively decrease the interface barriers and enhance electron extraction efficiency. Optical haze with cellulose has been proved to increase the light absorption of active layer for OSCs.^22^, ^23^ The optical haze and transmittance of these ITO/ZnO and ITO/ZnO/CMC films were measured through UV–vis–NIR spectrophotometry. As shown in Figure 1b, a transmission haze over 20% is found in these ITO/ZnO/CMC films annealed at 120 °C while maintaining a transmittance of ≈82% in the range of 370 to 900 nm, while the film of ITO/ZnO shows no haze in this regime with a transmittance of ≈84.5%. When the annealing temperature was increased to 150 °C for the film ITO/ZnO/CMC, the haze was decreased to be a value of ≈16%, and the transmittance remained almost the same with annealing at 120 °C. ITO/ZnO/CMC film annealed at 120 °C exhibited higher transmission haze and similar transmittance than that of others. This implies that the CMC film annealed at 120 °C will improve light absorption of solar cells from the increased transmittance of light into the active layer, thus leading to an enhanced short‐circuit current density (*J*_SC_).

**Figure 1:**
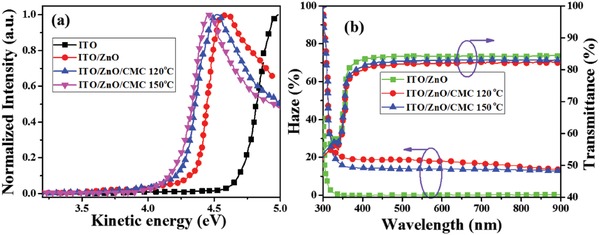
a) UPS spectra of bare ITO, ITO/ZnO (≈35 nm), and ITO/ZnO (≈35 nm)/CMC (≈8 nm) (annealed at 120 °C) and ITO/ZnO (≈35 nm)/CMC (≈8 nm) (10 nm) (annealed at 150 °C). b) The optical transmittance and haze versus wavelength measured with an integrating sphere setup.

The morphologies of ITO/ZnO and ITO/ZnO/CMC films were investigated by atomic force microscopy (AFM), as shown in **Figure** 2. It can be observed that the root‐means‐square (rms) roughness of pristine ZnO film is very small with the value of ≈1.432 nm, indicating the formation of smooth film of pristine ZnO on ITO surface. When the CMC is coated on the ZnO layer without annealing, a dramatic increase of surface roughness is found with the value of ≈6.358 nm (Figure 2c), the distinct nanophase structure of CMC is formed on ZnO, which will be good for improving transmission haze.^24^ Furthermore, the changes in surface morphologies are found largely after annealing at 120 °C and 150 °C (Figure 1d,f). The coverage density of the aggregates became sparse and the size of the aggregates became smaller, especially by annealing at 150 °C. The aggregates disappeared totally and a much flatter surface with nanosized particles was formed. The root mean square (RMS) value after annealing at 120 °C was 2.212 nm and after 150 °C was 1.328 nm. The smooth film is beneficial for charge extraction, and leads to an increase in *J*_SC_ as well as the device efficiency.

**Figure 2:**
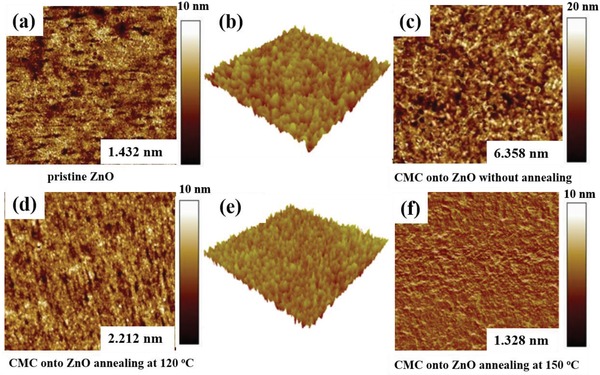
a) AFM image of ZnO film, b) 3D surface plot of pristine ZnO, c) AFM image of CMC film on ZnO without annealing, d) AFM image of CMC film on ZnO annealing at 120 °C, e) 3D surface plot of CMC film on ZnO annealing at 150 °C, and f) AFM image of CMC film on ZnO annealing at 150 °C.

The inverted OSCs were fabricated with the configuration of ITO/ZnO/CMC/PBDB‐T:IT‐M/MoO*_x_*/Ag by using ZnO and CMC as interface layers. In contrast approaches, devices with pristine ZnO were also prepared. The current density–voltage (*J*–*V*) and external quantum efficiency (EQE) curves are shown in **Figure** 3 and important parameters are summarized in **Table** 1. The pristine ZnO‐based device shows a *J*_SC_ of 16.57 mA cm^−2^, an open‐circuit voltage (*V*_OC_) of 0.935 V, fill factor (FF) of 70.83%, and a PCE of 10.97%, which is in agreement with the reported results.^25^ By depositing CMC onto ZnO layer and annealing at 120 °C, a positive effect on the performance of OSCs was observed. The PCE was enhanced to 12.01% from 10.97% (without CMC) with the *V*_OC_ of 0.935 V, *J*_SC_ of 17.87 mA cm^−2^, and FF of 71.87%, respectively. Similarly, when the ITO/ZnO/CMC layer was annealed at 150 °C, a high PCE of 11.63% was obtained with the *V*_OC_, *J*_SC_, and FF of 0.932 eV, 17.48 mA cm^−2^, and 71.41%, subsequently. Although the ITO/ZnO/CMC device annealed at 150 °C exhibited less PCE (11.63%) compared to the ITO/ZnO/CMC device annealed at 120 °C (12.01%), but still the PCE was significantly higher (5.7%) than that of the control device (10.97%). Among all the devices, the devices with ITO/ZnO/CMC (120 °C annealed) demonstrated the best performance. For these devices with CMC layer, the *J*_SC_ was increased significantly, especially for the device with CMC (120 °C annealed). An enhanced *J*_SC_ of 17.87 mA cm^−2^ was observed, which was ≈7.8% higher than that of pristine ZnO‐based devices, resulting an improvement in PCE by 9.4% (12.01% versus 10.98%). The increase in *J*_SC_ was ascribed to the enhanced light absorption by the active layer. All of the devices by employing CMC as interlayer showed better performance than that of pristine ZnO‐based ones, indicating a strong potential of CMC layer for the interfacial modification of OSCs.

**Figure 3:**
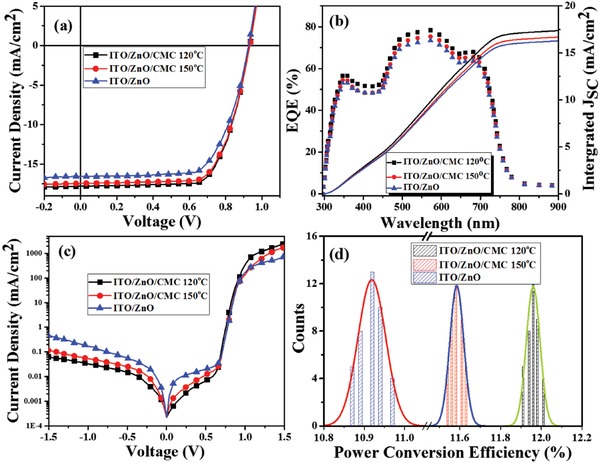
a) *J*–*V*, b) EQE with integrated currents, and c) dark *J*–*V* curves of ITO/ZnO, ITO/ZnO/CMC annealing at 120 °C, and ITO/ZnO/CMC annealing at 150 °C. d) Histogram of device efficiencies based on the 40 devices fabricated independently.

**Table 1:** Some parameters for OSCs with or without interfacial materials of CMC (device area: 4.0 mm^2^)

Interface layer	*J*_SC_ [mA cm^−2^]	*V*_OC_ [V]	FF [%]	PCE^avg^ ^a)^ [%]	PCE^best^ [%]
ZnO	16.57 ± 0.02	0.935 ± 0.005	70.85 ± 0.01	10.92 ± 0.05	10.97
ZnO/CMC(120 °C annealed)	17.87 ± 0.02	0.935 ± 0.005	71.87 ± 0.01	11.96 ± 0.05	12.01
ZnO/CMC(150 °C annealed)	17.48 ± 0.02	0.932 ± 0.005	71.41 ± 0.01	11.58 ± 0.05	11.63

^a)^ The average PCE was derived from 40 parallel devices.

The EQE curves of these OSCs with/without CMC layer are presented in Figure 3b. The EQE based on CMC layer (120 °C annealed) shows a maximum value of ≈78.5% and ≈70% across the range of 300–800 nm, indicating highly efficient photoelectron conversion process. While the corresponding ZnO‐based device demonstrates an EQE of ≈62% in the range of 300–800 nm. It is clear that the EQE is significantly enhanced by inserting a CMC layer between the active layer and ZnO film. The results are consistent with the trend of the UV–vis absorption results as shown in the Figure S2 in the Supporting Information. The calculated *J*_SC_ from the integration of the EQE curves is 17.4 mA cm^−2^ (120 °C annealed), 16.71 mA cm^−2^ (150 °C annealed), and 16.3 mA cm^−2^ (pristine ZnO), which is a less than ≈5% mismatch than that of the *J*_SC_ value from the *J*–*V* curves. The present mismatch is from the difference of spectrum, measured environment, the size of light spot, and flatness of the sample. Generally, the *J*–*V* curves are tested under a simulated sunlight with full solar spectrum, while their EQEs are measured from a single wavelength light from 300 to 900 nm with an interval of 10 nm. On the other hand, the measured environment was different for EQE and *J*–*V* characteristics. The EQEs were measured in air without encapsulation, while *J*–*V* curves were obtained in the inner box. The water and oxygen in air would lead to the decreased EQE. The high EQE in the devices with CMC layer (120 °C annealed) was from the increased absorption provided by the promoted transmission haze of the CMC layer. Dark *J*–*V* characteristic was also tested which provided some important information for the changes of these interface layers. As it can be seen in Figure 3c that the leakage current of ZnO with CMC layer devices is reduced in the forward‐biased and reverse‐biased regions, while the ZnO‐based devices show higher leakage current than that of CMC modified one. It should be pointed out that leakage current of ZnO with CMC annealed at 120 °C is lower than that of annealed at 150 °C. It can be explained to be the larger roughness of CMC annealed at 120 °C, which can increase the contact and decrease the traps between CMC and active layers.^26^ In the high voltage regime, the series resistance depends on the dark current. The higher current densities reflect that the involved CMC layer decreased the series resistance significantly, therefore, the electron transport and collection was enhanced at the interface of electrode and active layer. Pristine ZnO‐based devices show the lowest injection current among these devices, suggesting poor electron injection and diode rectification effects.^27^ A histogram of 40 devices fabricated independently (**Figure** 4d) shows excellent reproducibility with an average PCE of 11.96% for ITO/ZnO/CMC annealing at 120 °C. An increment in PCE is ascribed to the enhancement in *J*_SC_, whereas *V*_OC_ and FF remain almost the same.

**Figure 4:**
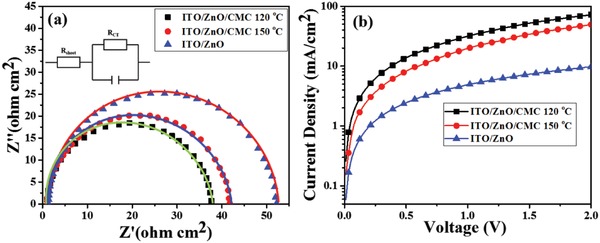
a) Nyquist plots and b) electron‐only device *J*–*V* curves of ITO/ZnO, ITO/ZnO/CMC annealing at 120 °C, and ITO/ZnO/CMC annealing at 150 °C.

The effects of ITO/ZnO, ITO/ZnO/CMC annealing at 120 °C, and ITO/ZnO/CMC annealing at 150 °C were investigated to elucidate the charge transfer and collection mechanism. As shown in the inserted image of Figure 4a, impedance spectra of ITO/ZnO, ITO/ZnO/CMC annealing at 120 °C, and ITO/ZnO/CMC annealing at 150 °C were obtained with the model of potentiostat on ZENNIUM Zahner equipment. The charge transport resistance (*R*_CT_) is related to the active layer/electrode interfaces and the active layers, the corresponding series resistance (*R*_S_) contains the sheet resistance (*R*_sheet_) of the electrodes.^28^ In the Figure 4a, the *R*_CT_ values of these devices with the active layer of PBDB‐T:IT‐M with or without CMC layer at different annealing temperature are 37.79 (ITO/ZnO/CMC annealing at 120 °C), 41.88 (ITO/ZnO/CMC annealing at 150 °C), and 52.44 (ITO/ZnO) Ω cm^2^, respectively. Among all devices, *R*_CT_ value of CMC‐based device annealing at 120 °C is lowest, indicating the improved the electron transfer and collection in these OSCs. In comparison, the device with pristine ZnO layer exhibited a slight larger *R*_CT_ value than that of the others. This reveals that CMC layer has played a decisive role to improve the charge transfer and collection, which is in line with the reduced properties of the dark *J*–*V* characteristics.

The electron mobilities of ITO/ZnO and ITO/ZnO/CMC devices annealed at 120 and 150 °C were measured by the method of space‐charge limited current. Electron‐only device was fabricated with the structure of Al/ZnO/with or without CMC/PBDB‐T:IT‐M/Ca/Al. As shown in Figure 4b, the devices with CMC layer show enhanced current densities, confirming the improved electron transport properties of the CMC interface. Particularly, the current density of the electron‐only device based on the ZnO/CMC layer annealed at 120 °C is much higher than that of the device based on the pristine ZnO layer, indicating better electron transportation. The result is consistent with the higher performance of the devices based on ZnO/CMC cathode annealed at the 120 °C (Figure 3a), Nyquist plot analysis (Figure 4a), and the enhanced dark current under voltages regime in the dark *J*–*V* curves (Figure 2c). To confirm the enhanced electron transfer through from CMC incorporation, other electron‐only devices with the configurations of Al/ZnO and CMC/Ca/Al were made to find the electron mobilities of ZnO and CMC, respectively (Figure S3, Supporting Information). The electron mobilities of CMC with different annealing temperatures are almost two order of magnitude lower than that of ZnO (4.68 × 10^−7^ cm^2^ V^−1^ s^−1^ for CMC annealing at 120 °C and 7.27 × 10^−5^ cm^2^ V^−1^ s^−1^ for ZnO), which is inconsistent with the results from the electron‐only devices of ZnO/CMC/PBDB‐T:IT‐M and ZnO/PBDB‐T:IT‐M (Figure 4a). The distinct results reveal that the changed interfacial contact from organic–inorganic contact of ZnO/IT‐M to organic–organic contact of CMC/IT‐M, is in favor of the electron transfer at the interface of the ZnO/CMC‐based device.

In conclusion, a facile and effective approach was developed to prepare cellulose and its derivatives as interfacial materials for efficient OSCs from rice straw agroforestry residues. By using these CMCs as interfacial modifiers at different annealing temperature, the enhanced PCEs and current densities in inverted OSCs were observed. The highest PCE of 12.01% for inverted OSC based on PBDB‐T:IT‐M as the active layer was obtained with a *V*_OC_ of 0.935 V, *J*_SC_ of 17.87 mA cm^−2^, and FF of 71.87%, which shows over 9.4% improvement compared to that of the device without the CMC layer (10.98% PCE, *V*_OC_ of 0.935 V, *J*_SC_ of 16.57 mA cm^−2^, and FF of 70.85%). The current densities and PCEs were enhanced in these devices with CMC modified layer. The improved current densities and PCEs are attributed to the decreased work function, increased absorption, and interfacial contact by using CMC and ZnO as co‐interface. Our findings will provide a new way to develop new interlayer materials from agroforestry residues with improved efficiency for green organic electronic devices.

## Conflict of Interest

The authors declare no conflict of interest.

## Supporting information

Supporting Information
