# Supplementary material for: From Straw to Device Interface: Carboxymethyl‐Cellulose‐Based Modified Interlayer for Enhanced Power Conversion Efficiency of Organic Solar Cells
Source: Adv Sci (Weinh). 2019 Nov 27;7(2):1902269. doi: 10.1002/advs.201902269 (PMC6974931; doi:10.1002/advs.201902269)
Supplement: Supplementary file 1 — Supporting Information [file ADVS-7-1902269-s001.pdf]

Copyright WILEY-VCH Verlag GmbH & Co. KGaA, 69469 Weinheim, Germany, 2019.

ADVANCED SCIENCE
Open Access

Supporting Information

for *Adv. Sci.,* DOI: 10.1002/advs.201902269

From Straw to Device Interface: Carboxymethyl-Cellulose-Based Modified Interlayer for Enhanced Power Conversion Efficiency of Organic Solar Cells

*Junying Wu, Yanjun Liu, Amjad Islam, Qinghong Zheng, Jianguo Li, Wei Ji, Lihui Chen, and Xinhua Ouyang**

From straw to device interface: carboxymethyl cellulose based modified interlayer for enhanced power conversion efficiency of organic solar cells

*Junying Wu,Yanjun Liu, Amjad Islam, Qinghong Zheng, Jianguo Li, Wei Ji, Lihui Chen, and Xinhua Ouyang**

Experimental Section

1. Preparation of carboxymethyl cellulose

1.1 Materials and Characterization

Dried rice straw was from Shaowu county of Fujiang province, which was cut into ~3 cm segments. isopropyl alcohol, sodium hydroxide, sodium chloroacetate, and methanol were purchased from Sinopharm Chemical Reagent Co. Ltd. They were used without purification. FT-IR were tested on a Nicolet 380 spectrometer (VERTEX 70, Bruke, Germany). pH value was measured on Mettler Toledo Seven Excellence.

1.2 Preparation of carboxymethyl cellulos

1 KG rice straw was cooked by sulfate method in a 15 L heating cooker1. They are cooked at 120 ^{o}C with 2% NaOH solution and the weight ratio of 1:7 (straw and NaOH solution). After 2 h, the mixture was cooled and gotten a softwood straw pulp. Then, 5 g straw pulp was added 150 mL isopropyl alcohol, 10 mL 30% NaOH, and 4.5 g sodium chloroacetate, stirring at 60 ^{o}C for 4h. The insoluble substance was collected after centrifugation and washed three times with methanol-water (9:1, V/V), dried it in vacuum for 24 h. Yield, 2.54 g, 50.8%.

1.3 Characterization of degree of substitution (DS)

0.2 g sample was dissolved in distilled water of 80 mL, stirred for 10 minutes, the pH value of the solution was controlled to be 8, then titrated with standard sulfuric acid

solution in beaker with acidity meter electrode, and observed the changes of acidity indicator value to be 3.74. Then, recorded the used volume of standard sulfuric acid solution. By using the formula of DS=0.162B/(1-0.08B), the value of DS was 0.99, where B is $2M(V_2-V_1)/m$.

1.4 FT-IR spectra

As shown in the Figure S1, the absorption peaks at 3200-3500 cm^{-1} were from hydroxyl group and water in rice straw cellulose. Importantly, the peaks l ^{o}C ated at 1599 cm^{-1} and 1416 cm^{-1} were from the carbonyl asymmetric stretching vibration and symmetric stretching vibration of -COONa in CMC respectively, which indicated that the cellulose molecules had successfully reacted with chloroacetic acid, and some hydroxyl hydrogen in the molecule was replaced by carboxymethyl. The absorption peaks at 1008 cm^{-1} and 825 cm^{-1} belong to the stretching vibration of C-O and C-H on cellulose molecule.

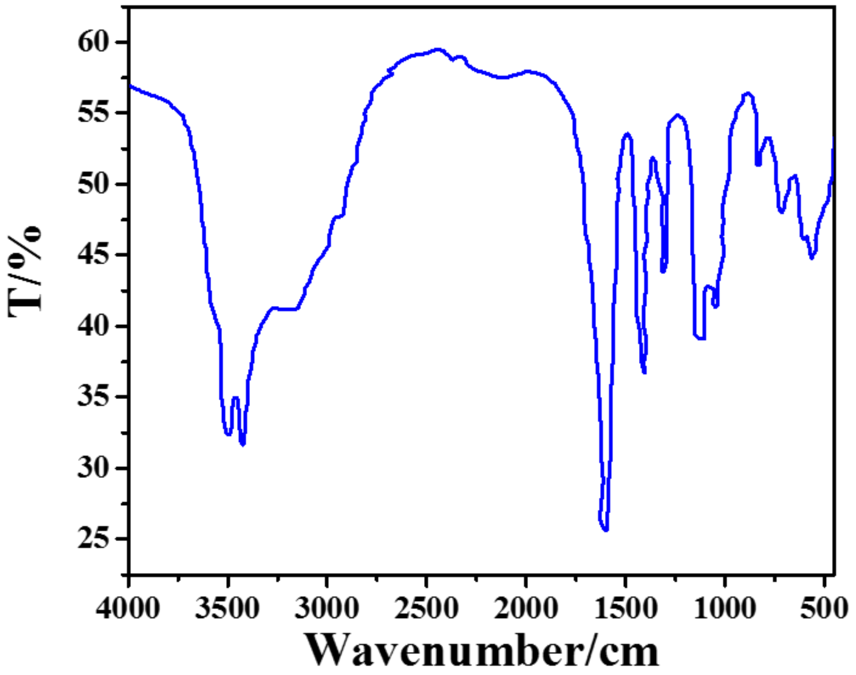

Figure S1. FT-IR spectra of carboxymethyl celluloseigure Caption.

1.5 Absorption of blend film with or without CMC

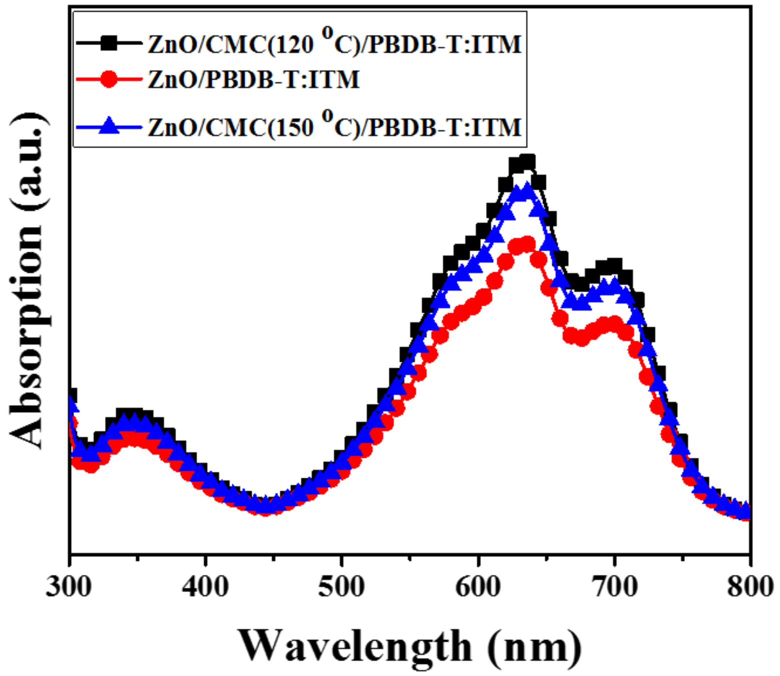

Figure S2. Absorption spectra of blend film with or without CMC

In order to get rid of the effect of CMC layer, we prepared the active-layer on CMC and ZnO, respectively. Their thickness is tested by profilometer and the results were summarized in Figure S3. As shown in the figure, there is little change in the film thickness of the active layer before and after CMC spin coating (all of which are about 100nm).

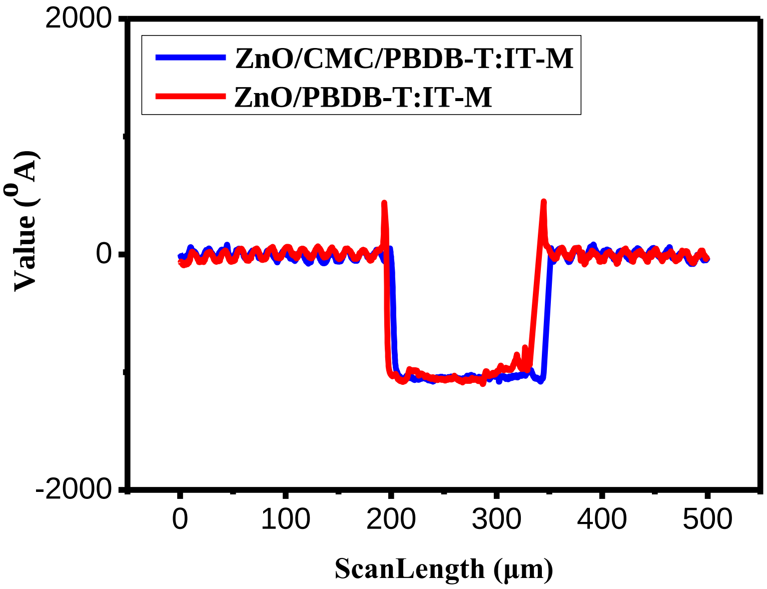

Figure S3. Film thickness of the active layer with or without CMC.

1.6 Resistance of CMC in deposition active layer organic solvent (CB).

As can be seen in Figure S4, it can be seen the CMC is not dissolved in the solvent of CB, which is used to dissolve active layer organic solvent with the concentration of 0.1 mg/mL. Obviously, the CMC layer can be inferred to be resistant to the organic solvent used to deposit the active layer.

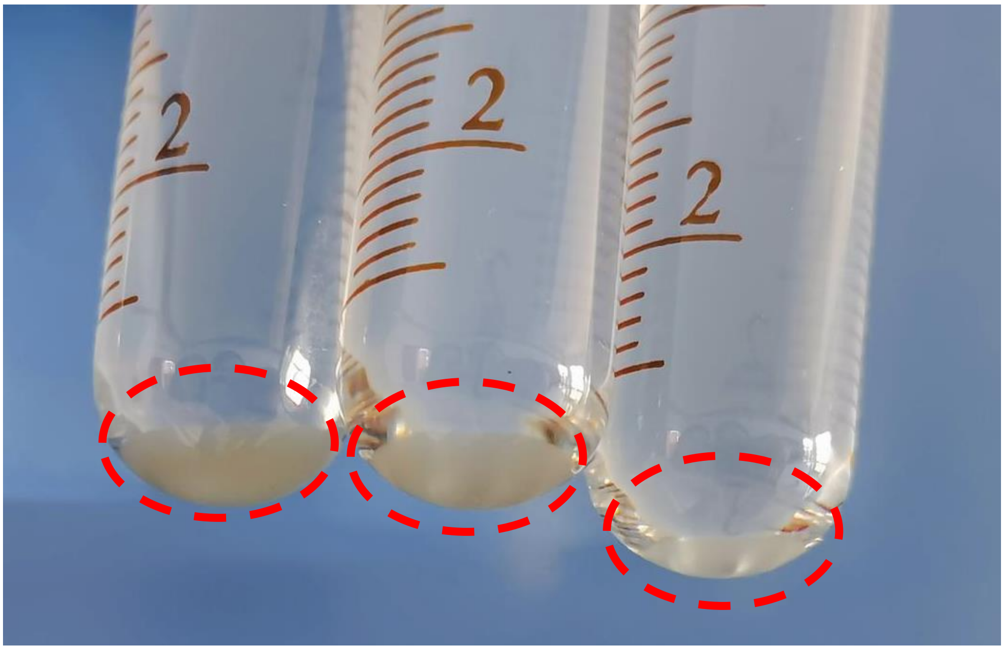

Figure S4. CMC solution of different concentration, the solvent is CB.

2. Device fabrication and measurement

2.1 Device fabrication

The indium-doped tin oxide glass sheet (0.7 mm thick, ≤15Ω/square, transmittance > 90%) was purchased from Jinghui Science and Techlogy Company, Ltd. They were cleaned by using sonication in detergent, deionized water, acetone, and isopropyl alcohol and dried in a vacuum funace, Then, by an UV-ozone treated them by UV-ozone plasma about 10 minutes. The ZnO precursor solution was prepared by dissolving 0.4 g of zinc acetate dehydrate and 0.112 g of ethanolamine in 4 mL of 2-methoxyethanol. ZnO precursor solution was spin-coated on top of ITO glass at 3500 rpm for 40 s following a thermal annealing at 200 ^{o}C for 30 min in air.

Subsequently, the CMC was dispersed in a mixed solution of methanol (90%) and water (10%), and spin-coated on the ZnO layers with different annealed temperature. The speed of coater is 2700 rpm for 60 s with the thickness of 8 nm. After the deposition, the substrate was transferred to the Ar-filled glovebox. The materials PBDB-T:IT-M were dissolved in CB with the total concentration of 20 mg/ml^{-1} and stirred at 50 ^{o}C for 12 hours. Then, the mixture were spin coated on CMC modified layer at 1600 rpm to the optimal film thickness of ~100 nm. The active layers were then thermal annealed for 10 minutes at 160 ^{o}C. Finally, 5 nm thick MoO_3 film and 100 nm thick Al layer were deposited sequentially to complete the inverted device. The device area was defined to 0.04 cm ^{2}.

2.2 J-V characterization and EQE measurement

The J-V characteristics of OSCs were measured in an Ar-filled glovebox using a Keithley 2400 source-measure unit and an AM 1.5 G solar simulator (Newport-Oriel® Sol3A 450W). The illumination intensity of 100 mW cm^{-2} irradiation was calibrated using a standard monocrystal Si reference cell (PV measurements Inc.) to ensure the accurate light source intensity.The EQE was carried out on a Newport-Oriel®OPS-A500, which was calibrated by standard Si/Ge solar cell under illumination with monochromatic light from a Xe lamp at room temperature in air.

3. Electron-only device study

Electron-only devices were fabricated for the studies of electron mobility by using space charge limited current (SCLC) method. The device structures are Al/ZnO/ with or without CMC/PBDB-T:IT-M/Ca/Al and Al/ZnO or CMC/Ca/Al, respectively.

The SCLC is described by modified Mott-Gurney's law[3]:

$J=(9/8)\varepsilon_0\varepsilon_r\mu(V^2/d^3)\exp[0.89\beta(V/d)^{0.5}]$

We can get the following formula:

$\ln(Jd^3/V^2)\approx0.89\beta(V/d)^{0.5}+\ln(9\varepsilon_0\varepsilon_r\mu/8)$

where J stands for current density, d is the thickness of the active layer, V is the applied potential, εr is the relative dielectric constant of the blend (assuming that 3.5), ε_0 is the permittivity of free space (8.85×10^{-12} C $V^{-1}s^{-1}$), β is the field activation factor and m is the electron mobility.

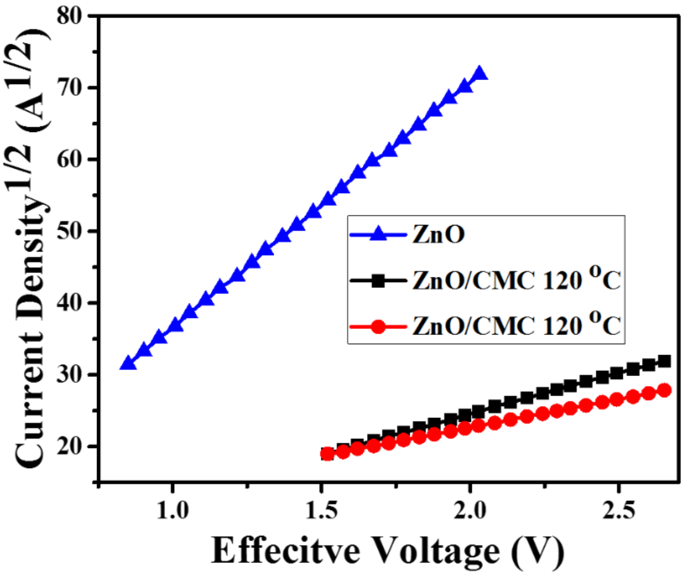

Figure S5 . $J^{1/2}\sim V$ characteristics of electron-only devices with configuration of Al/ZnO or CMC/Ca/Al.

As shown in the Table S1, the mobilities of Al/CMC (100 nm)/Ca (20 nm)/Al, Al/ZnO (100 nm)/Ca (20 nm)/Al, Al/ZnO (20 nm)/PBDB-T:IT-M (100 nm)/Ca (20 nm)/Al, and Al/ZnO (40 nm)/CMC (8 nm)/PBDB-T:IT-M (100 nm)/Ca (20 nm)/Al had been investigated in detail. For pristine Al/CMC (100 nm)/Ca (20 nm)/Al, the thickness of CMC is ~100 nm, the electron tunnel only a little from electrodes Al to Ca (20 nm)/Al due to the limitation of electron diffusion length (10~20 nm). Thus, the

measured electron mobility of Al/CMC (100 nm)/Ca (20 nm)/Al is low with the value of several 10^{-7} cm^2 V^{-1} s^{-1}. While the mobilities for the devices of Al/ZnO (40 nm)/CMC (8 nm)/PBDB-T:ITM (100 nm)/Ca (20 nm)/Al are significantly increased as the synergetic effect of ZnO, CMC, and PBDB-T:ITM. It should be pointed out that the thickness of CMC is changed to be 8 nm, which is in the regime of electron diffusion length. The electron can migrate easily from ZnO to PBDB-T:IT-M. Comparing with the result of Al/ZnO (20 nm)/PBDB-T:IT-M (100 nm)/Ca (20 nm)/Al, the mobilities are the same order of magnitude. A little improvement is attributed to the changed interfacial contact from organic-inorganic contact of ZnO//PBDB-T:IT-M to organic-organic contact of CMC//PBDB-T:IT-M, which has been demonstrated by some of publications .

Table S1. SCLC mobilities of Al/ZnO/ with or without CMC/PBDB-T:IT-M/Ca/Al and Al/ZnO or CMC/Ca/Al

Device structure	Mobilities
Al/ CMC (120°C)/Ca/Al	4.68×10^{-7} cm^2 V^{-1} s^{-1}
Al/ CMC (150°C)/Ca/Al	2.73×10^{-7} cm^2 V^{-1} s^{-1}
Al/ ZnO/Ca/Al	7.27 $\times10^{-5}$ cm^2 V^{-1} s−1
Al/ZnO/PBDB-T:IT-M/Ca/Al	2.29 $\times10^{-4}$ cm^2 V^{-1} s^{-1}
Al/ZnO/CMC (120°C)/PBDB-T:IT-M/Ca/Al	3.96 $\times10^{-4}$ cm^2 V^{-1} s^{-1}
Al/ZnO/CMC (150°C)/PBDB-T:IT-M/Ca/Al	3.24 $\times10^{-4}$ cm^2 V^{-1} s^{-1}

Reference

1. Rong Hee Lee *et al*, *Cellulose* 2019, .org/10.1007/s10570-019-02724-2).
